# Gallbladder carriage generates genetic variation and genome degradation in *Salmonella* Typhi

**DOI:** 10.1371/journal.ppat.1008998

**Published:** 2020-10-21

**Authors:** Pham Thanh Duy, Nga Tran Vu Thieu, To Nguyen Thi Nguyen, Ho Ngoc Dan Thanh, Sabina Dongol, Abhilasha Karkey, Megan Carey, Buddha Basnyat, Gordon Dougan, Maia A. Rabaa, Stephen Baker

**Affiliations:** 1 The Hospital for Tropical Diseases, Wellcome Trust Major Overseas Programme, Oxford University Clinical Research Unit, Ho Chi Minh City, Vietnam; 2 Centre for Tropical Medicine, University of Oxford, Oxford, United Kingdom; 3 Oxford University Clinical Research Unit, Patan Academy of Health Sciences, Kathmandu, Nepal; 4 Cambridge Institute of Therapeutic Immunology & Infectious Disease (CITIID) Department of Medicine, University of Cambridge, Cambridge, United Kingdom; Stanford University School of Medicine, UNITED STATES

## Abstract

Despite recent advances in typhoid fever control, asymptomatic carriage of *Salmonella* Typhi in the gallbladder remains poorly understood. Aiming to understand if *S*. Typhi becomes genetically adapted for long-term colonisation in the gallbladder, we performed whole genome sequencing on a collection of *S*. Typhi isolated from the gallbladders of typhoid carriers. These sequences were compared to contemporaneously sampled sequences from organisms isolated from the blood of acute patients within the same population. We found that *S*. Typhi carriage was not restricted to any particular genotype or conformation of antimicrobial resistance genes, but was largely reflective of *S*. Typhi circulating in the general population. However, gallbladder isolates showed a higher genetic variability than acute isolates, with median pairwise SNP distances of 21 and 13 SNPs (*p* = 2.8x10^-9^), respectively. Within gallbladder isolates of the predominant H58 genotype, variation was associated with a higher prevalence of nonsense mutations. Notably, gallbladder isolates displayed a higher frequency of non-synonymous mutations in genes encoding hypothetical proteins, membrane lipoproteins, transport/binding proteins, surface antigens, and carbohydrate degradation. Specifically, we identified several gallbladder-specific non-synonymous mutations involved in LPS synthesis and modification, with some isolates lacking the Vi capsular polysaccharide vaccine target due to the 134Kb deletion of SPI-7. *S*. Typhi is under strong selective pressure in the human gallbladder, which may be reflected phylogenetically by long terminal branches that may distinguish organisms from chronic and acute infections. Our work shows that selective pressures asserted by the hostile environment of the human gallbladder generate new antigenic variants and raises questions regarding the role of carriage in the epidemiology of typhoid fever.

## Author summary

*Salmonella* Typhi is the bacterium that causes typhoid. *Salmonella* Typhi is infamous for being able to be carried in the gallbladder, with Typhoid Mary being the best-known example of a typhoid carrier. Despite having new tools for typhoid control, we have made little progress in understanding this disease process. Aiming to understand if *Salmonella* Typhi is adapted for long-term survival in the gallbladder, we sequenced the genomes of 24 *Salmonella* Typhi isolated from the gallbladders of typhoid carriers. We compared these genomes to *Salmonella* Typhi from acute typhoid patients within the same population. The carriage of *Salmonella* Typhi was not restricted to any specific genotype or resistance to antibiotics, but reflective of the organisms causing acute disease. However, gallbladder isolates had higher genetic variability than acute isolates, with a higher frequency of mutations changing the amino acid sequences of hypothetical proteins, membrane lipoproteins, transport/binding proteins, surface antigens, and carbohydrate degradation. We identified several gallbladder-specific mutations involved in polysaccharide synthesis on the bacterial surface. Our work shows that selective pressures asserted by the hostile environment of the human gallbladder generates genetic variation, which is not observed in acute isolates, raising questions regarding the role of carriage in the epidemiology of typhoid.

## Introduction

Typhoid fever, a life-threatening systemic infection caused predominantly by the bacterium *Salmonella enterica* serovar Typhi (*S*. Typhi), remains a significant public health problem in resource-poor settings including parts of Asia and Africa [1]. The disease is contracted via ingestion of contaminated food or water or through contact with individuals excreting the organism [2]. The majority of typhoid patients fully recover with appropriate treatment; however, some individuals can become asymptomatic carriers and shed infectious bacteria in their faeces for an ill-defined period of time. Asymptomatic carriage of *S*. Typhi has been recognized as a public health threat for more than a century, with infamous typhoid carriers like Mary Mallon, a cook in New York, and Mr N, a milker in England, identified in the early part of the 20^th^ century [3,4].

Typhoid carriage can be differentiated into three categories depending on the duration of shedding: convalescent (three weeks to three months), temporary (three to twelve months), and chronic (more than one year) [5]. In endemic regions, an estimated 2–5 percent of acute typhoid patients become chronic carriers, meaning that they continue to intermittently shed the bacteria indefinitely after apparent clinical resolution [3,5]. Consequently, chronic carriers are widely believed to be an ecological niche that facilitates the transmission and persistence of typhoid in human populations [6]. *S*. Typhi is a human-restricted pathogen, meaning that the disease may be theoretically eliminated locally by reducing transmission through targeted treatment, improved sanitation, and mass vaccination. Consequently, understanding the role of chronic carriers in disease transmission, and detecting them prospectively, may accelerate disease elimination.

Despite substantive gains in understanding the biology of typhoid, we have generated limited new insights into typhoid carriage in recent decades. Data from murine models of *Salmonella* carriage and human clinical investigations have determined that the gallbladder is a key permissive niche for long-term bacterial persistence [7–13]. Various epidemiological investigations have shown that gallstones and gallbladder damage may facilitate typhoid carriage [9,13–17], and that *Salmonella* preferentially attach to and form biofilms on cholesterol-rich gallstones [7,11,13,18,19]. *S*. Typhi carriage isolates have been previously genetically compared to isolates from acute infections, with the aim of identifying signatures associated with carriage [20–23]. However, these studies were unable to infer how carriage isolates directly relate to those causing acute disease.

It is apparent we need a better understanding of the role of the typhoid carrier and associated organisms to generate new approaches to the management of such individuals in endemic locations. Although it is widely accepted that that *S*. Typhi carriage plays a key role in the transmission of typhoid in endemic settings, it is unknown if carriage organisms are somehow adapted for long-term colonisation. Aiming to address this question, we performed whole genome sequencing and detailed genetic analyses of *S*. Typhi isolated from the gallbladders of typhoid carriers in Kathmandu. We compared these data to the sequences of contemporaneous organisms isolated from the blood of acutely infected patients in the same community over the same time period. Our data show that whilst carriage isolates are reflective of the general *S*. Typhi population circulating in the community, selective pressures during gallbladder carriage induce increased genetic variation and genomic degradation.

## Results

### The phylogenetic relationships between acute and gallbladder S. Typhi isolates

Between June 2007 and October 2010, we conducted a *Salmonella* carriage study in Kathmandu[13]. Patients undergoing cholecystectomy for acute or chronic cholecystitis were enrolled; bile and stool samples from 1,377 individuals were collected and subjected to microbiological examination. Twenty-four *S*. Typhi were isolated from bile samples taken from these patients and designated as gallbladder isolates. Ninety-six *S*. Typhi isolates recovered from patients with acute typhoid fever living in the same population over the same time period were used for comparison [24] (denoted as acute isolates) (S1 Table). A phylogenetic analysis of these 120 *S*. Typhi isolates demonstrated that subclade 4.3.1 (H58) was the dominant genotype, constituting 62.5% (15/24) of all gallbladder isolates and 65.6% (63/96) of all acute isolates. The second most common genotype was 3.3.0 (H1), accounting for 12.5% (3/24) and 14.6% (14/96) of all gallbladder and acute isolates, respectively.

**Fig 1:**
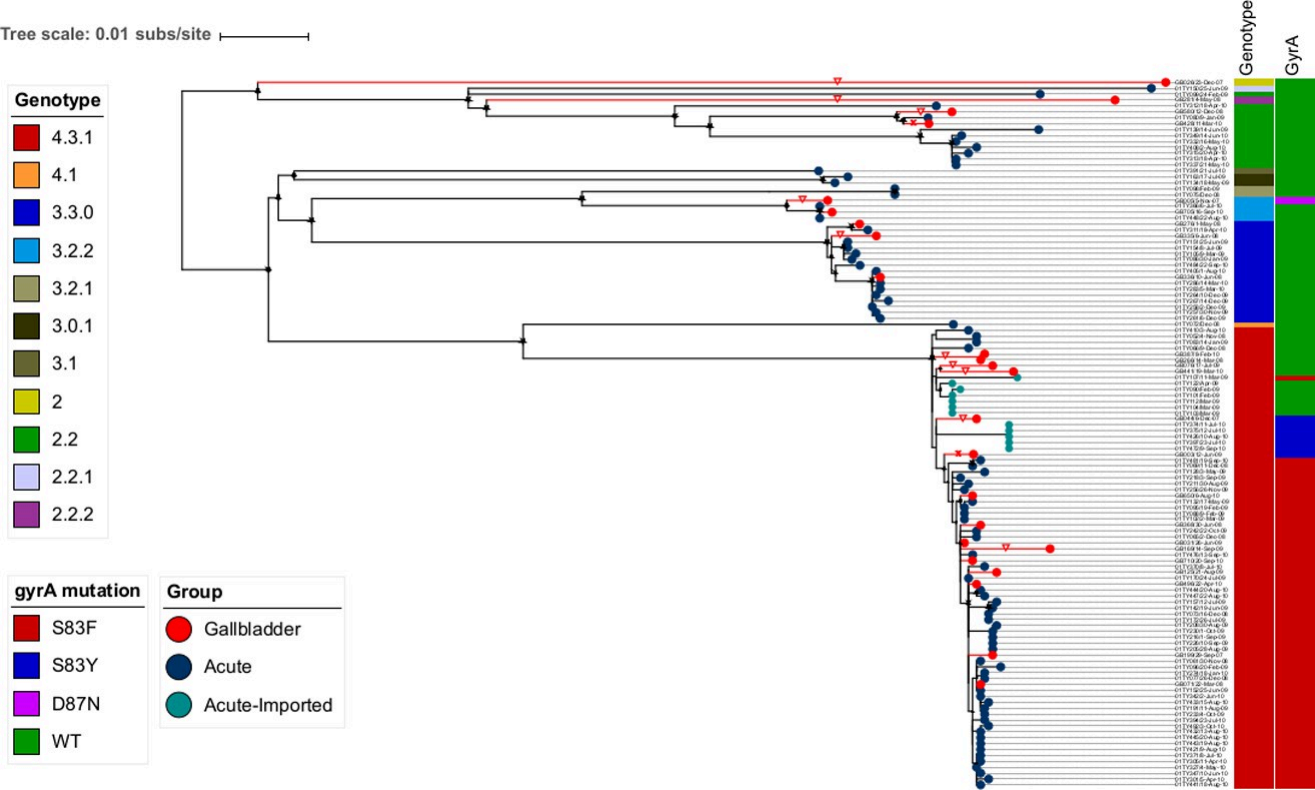
The phylogenetic structure of gallbladder and acute *S*. Typhi isolates collected between 2007 and 2010. Rooted maximum likelihood tree (*S*. Paratyphi A used as an outgroup to root the tree and pruned for visualization) based on core-genome SNPs of 120 *S*. Typhi isolates with the corresponding metadata: genotype, *gyrA* mutation. Gallbladder and acute isolates are shown as red and dark circles at the terminal nodes, respectively. Acute isolates originating from importation are also highlighted by turquoise circles at the terminal nodes. Terminal branches leading to gallbladder isolates are highlighted in red. Red triangles show gallbladder isolates associated with unusually long terminal branches.

We identified a significant degree of genetic diversity within this collection of acute and carriage organisms, with multiple less-common genotypes co-circulating, included various clades (4.1, 3.1 and 2.2), subclades (3.2.2, 3.0.1, 2.2.2 and 2.2.1), and organisms within primary cluster 2 (Fig 1). The less common genotypes from the gallbladder fell within subclade 3.2.2 (8.3%; 2/24), 2.2.2 (4.2%; 1/24), clade 2.2 (8.3%; 2/24) and primary cluster 2 (4.2%; 1/24). Overall, gallbladder isolates were not significantly associated with subclade 4.3.1 in comparison with other genotypes (15/24 versus 9/24, *p* = 0.083; Chi-squared test). These initial observations indicate that *S*. Typhi carriage was not restricted to any particular *S*. Typhi genotype; instead, the genotype distribution among gallbladder isolates generally reflected a phylogenetic structure similar to that of the acute *S*. Typhi infections circulating in the community.

### Antimicrobial susceptibility

We speculated resistance to key antimicrobials may facilitate the development of carriage. However, we found that the *S*. Typhi gallbladder isolates did not carry any acquired AMR genes. However, chromosomal mutations associated with reduced susceptibility to fluoroquinolones were common. These fluoroquinolone resistance-associated mutations within the gallbladder organisms were more commonly observed in subclade 4.3.1 than in non-subclade 4.3.1 (73% (11/15) versus 11% (1/9), *p* = 0.01; Chi-squared test). We compared *gyrA* mutation profiles between acute and gallbladder isolates within subclade 4.3.1; 76.2% (48/63) of acute isolates and 60% (9/15) of gallbladder isolates carried the S83F mutation, 7.9% (5/63) of acute isolates and 13.3% (2/15) of gallbladder isolates carried the S83Y mutation, and 15.9% (10/63) of acute isolates and 26.7% (4/15) of gallbladder isolates had no *gyrA* mutation. Consequently, there was no significant difference (*p =* 0.327; Chi-squared test) in the presence of fluoroquinolone resistance-associated mutations between acute and gallbladder isolates within subclade 4.3.1.

### Phylogenetic signatures of long-term Salmonella Typhi carriage

Despite the acute and gallbladder *S*. Typhi isolates generally clustering within the same genotypes across the phylogeny, we observed that a substantial proportion of the gallbladder isolates had higher genetic variability, as illustrated by their placement at the tips of long terminal branches within the phylogeny (Fig 1). The median pairwise SNP distance of gallbladder isolates within subclade 4.3.1 was 21 SNPs (IQR: 12–24), which was significantly greater than that of the corresponding acute isolates (13 SNPs (IQR: 8–19 SNPs) (*p* = 2.8x10^-9^, Wilcoxon rank-sum test) (S2 Fig). Similarly, the median pairwise SNP distance of gallbladder isolates within subclade 3.3.0 (20 SNPs, IQR: 13–22 SNPs) was higher than that of acute isolates (13 SNPs, IQR: 4–15 SNPs) (*p* = 0.26, Wilcoxon rank-sum test).

We mapped the contemporary acute and gallbladder *S*. Typhi sequences onto the global *S*. Typhi phylogeny, which indicated that the majority of these Nepalese acute and gallbladder *S*. Typhi isolates fell within known domestic genotypes, with limited evidence of importation from other countries (S1 Fig). This observation suggests that the long terminal branches associated with gallbladder isolates were unlikely to be driven by the importation of these organisms from alternative countries.

We next estimated and plotted the nearest phylogenetic distances (NPDs) between each taxon and its nearest neighbour on the subclade 4.3.1 tree versus the year of isolation, the age of the individual from whom the organism was isolated, and the *gyrA* mutation profile. We hypothesized that the annual distribution of NPDs of *S*. Typhi acute isolates would represent the phylogenetic diversity (mutation accumulation) occurring annually via acute disease transmission and would be comparable over multiple years. Alternatively, we considered that *S*. Typhi in the gallbladder may develop characteristic adaptive mutations facilitating long-term persistence, causing them to gradually become increasingly distinct from contemporaneous acute isolates, leading to greater phylogenetic distances relative to their neighbours. In addition, given that all acute subclade 4.3.1 isolates here exhibited a *gyrA* mutation, the gallbladder subclade 4.3.1 isolates without a *gyrA* mutation were more likely to have colonized the gallbladder prior to nalidixic acid resistance becoming commonplace.

**Fig 2:**
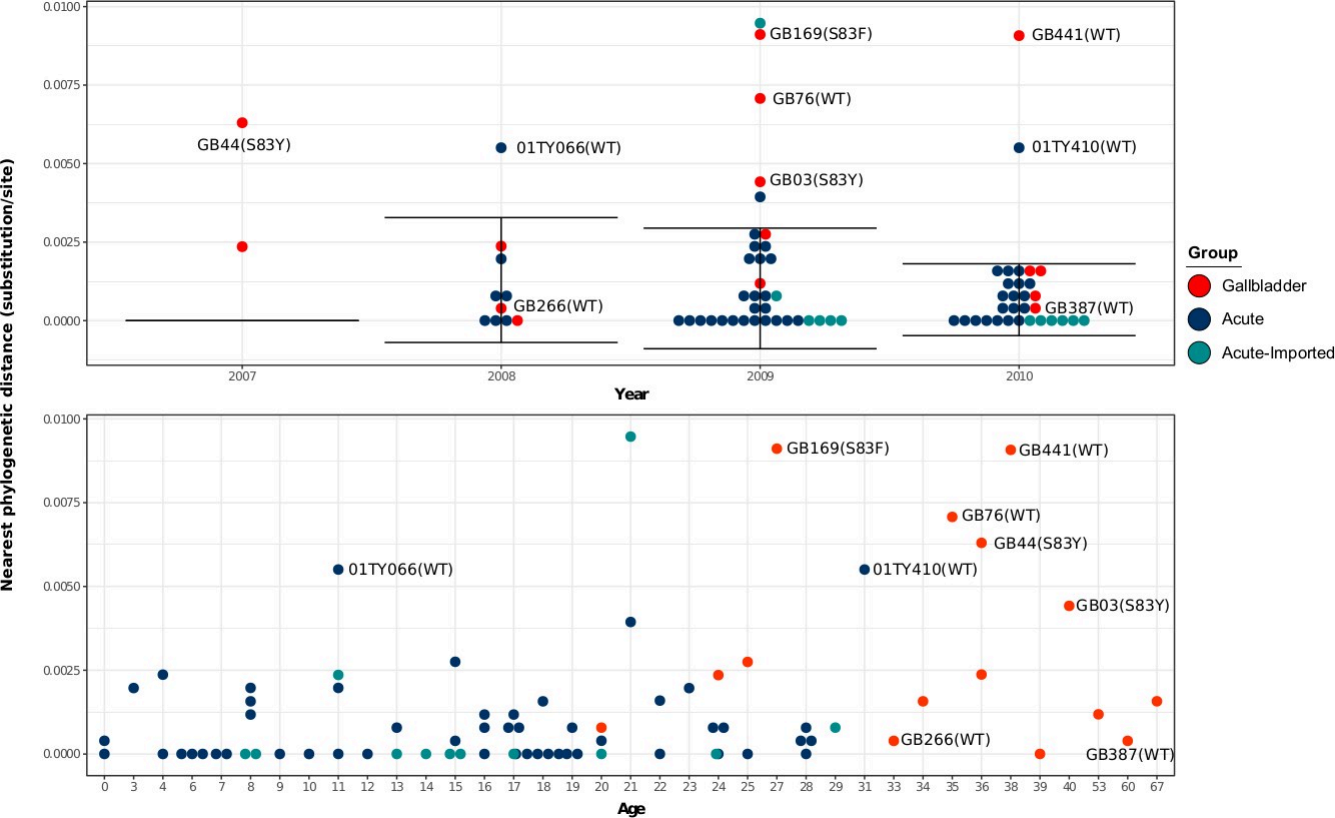
The Distribution of nearest phylogenetic distances of gallbladder and acute H58 isolates over the study period. Each circle represents the phylogenetic distance from each isolate to its nearest neighbour on the phylogenetic tree. The error bar represents the average phylogenetic distance to the nearest neighbour (± standard deviation) for acute H58 isolates. Gallbladder and acute isolates estimated to have originated from chronic carriers are labelled with their corresponding strain names.

Our analyses showed that the average (±SD) NPD per year of acute subclade 4.3.1 isolates was comparable; specifically, 0.00163 (± 0.00202) substitutions/site (~3.6 (± 4.4) SNPs) in 2008; 0.00110 (± 0.00229) substitutions/site (~2.4 (± 5) SNPs) in 2009, and 0.00144 (± 0.00238) substitutions/site (~3.2 (± 5.2) SNPs) in 2010. The majority of the subclade 4.3.1 gallbladder isolates (8/10) for which NPDs fell within the annual NPD distribution of acute subclade 4.3.1 isolates were associated with comparable terminal branch lengths and had a *gyrA* S83F mutation. Based on these findings, we surmised that gallbladder colonization with these isolates was likely to have occurred relatively recently in these individuals. Notable exceptions were two gallbladder isolates (GB266 and GB387) that did not possess a *gyrA* mutation and were associated with long terminal branches but had low NPDs as they clustered closely within the main phylogeny (Figs 1 and 2). Further, our data showed that all subclade 4.3.1 gallbladder isolates exhibiting abnormally high NPDs were associated with long terminal branches, suggestive of chronic carriage (Fig 2). In particular, two subclade 4.3.1 gallbladder isolates (GB76 and GB441) lacked *gyrA* mutations, two others (GB003 and GB044) had *gyrA* S83Y mutations, and the remaining one (GB169) exhibited *gyrA* mutation S83F. With respect to the age distribution, typhoid carriers were significantly older (median age 36 years, range: 20–67) than patients with acute illness (median age 16 years, range: 0–31) (*p* = 3.8x10^-8^, Wilcoxon rank-sum test). The gallbladder isolates thought to have originated from chronic carriers (based on above data) were obtained from individuals between aged between 27 and 40 years, which was older than the majority of the sampled acute typhoid patients; however, there was no significant difference in age distribution between those estimated to be recent and chronic carriers (Fig 2).

### The genetic traits of Salmonella Typhi gallbladder isolates

To identify potentially adaptive mutations associated with typhoid carriage, all nonsynonymous SNPs (NSs) occurring exclusively within the *S*. Typhi gallbladder genome sequences were identified and grouped by their predicted or known function. A corresponding analysis was performed for all NSs in the acute *S*. Typhi isolates. A total of 228 gallbladder-specific NSs (212 missense and 16 nonsense mutations) and 469 acute-specific NSs (437 missense and 32 nonsense mutations) were identified. In general, there was no significant difference (*p* = 0.924; Chi-square test) in the proportion of nonsense mutations out of total specific NSs in the gallbladder versus the acute isolates across all genotypes. However, among subclade 4.3.1 isolates, the proportion of nonsense mutations out of total specific NSs was significantly higher for gallbladder isolates than for acute isolates (10/60 compared to 2/67, Fisher exact test, *p* = 0.01). These data suggest that gene degradation resulting from nonsense mutations was more common in the subclade 4.3.1 gallbladder isolates compared to the subclade 4.3.1 acute isolates.

Inactivated genes in the gallbladder isolates included genes involved in the synthesis of peptidoglycan (*pbpC*), vitamin B12 receptor (*btuB*), general stress response regulator (*rpoS*), a laterally acquired protein in SPI-7 (STY4562), membrane transport protein (STY3932), central metabolism (STY0230, *ggt*), hypothetical proteins (STY0929 and STY4178), and osmotically inducible lipoprotein E precursor (*osmE*) (Table 1).

**Table 1:** Nonsense mutations and their predicted functions in gallbladder isolates.

Position in CT18	S/NS	Gene	Product	Functional class	GB005	GB026	GB044	GB076	GB125	GB169	GB199	GB266	GB281	GB335	GB368	GB387	GB441	GB580	GB705
239853‥241370	STOP	STY0230	Deoxyguanosine-triphosphate trophosphohydrolase	Central intermediary metabolism							E496*								
378398‥378796	STOP	STY0368	probable secreted protein	membrane lipoprotein									C46*						
598006‥600618	STOP	fimD	outer membrane usher protein FimD precursor	surface structure														Q386*	
1457973‥1458758	STOP	STY1502	probable secreted protein	membrane lipoprotein										W162*					
complement (1721748‥1722089)	STOP	STY1802	osmotically inducible lipoprotein E precursor	unknown						Q99*									
complement (2513933‥2514808)	STOP	STY2679	sulphate transport system permease protein CysW	transport anion		W175*													
complement (2629668‥2631983)	STOP	pbpC	pencillin binding protein 1C	murein sacculus peptidoglycan			Q246*												
complement (2915077‥2916069)	STOP	rpoS	RNA polymerase sigma subunit RpoS	broad regulatory function					W247*										
complement (3601247‥3603091)	STOP	STY3744	vitamin B12 receptor protein	cell envelope											W33*				
3795271‥3796734	STOP	STY3932	putative membrane transport protein	transport/binding protein					Q15*										
complement (4037181‥4038752)	STOP	STY4178	Conserved hypothetical protein	hypothetical protein											W413*				
4123472‥4125214	STOP	ggt	gamma-glutamyltranspeptidase precursor	thioredoxin											Q105*				
4307686‥4308996	STOP	STY4438	putative exported protein	membrane lipoprotein														W184*	
4442121‥4444211	STOP	STY4562	hypothetical protein	SPI-7								W234*				W234*			
4593908‥4595071	STOP	yjfC	conserved hypothetical protein	hypothetical protein		W9*													

* mutations generating a stop codon

**Fig 3:**
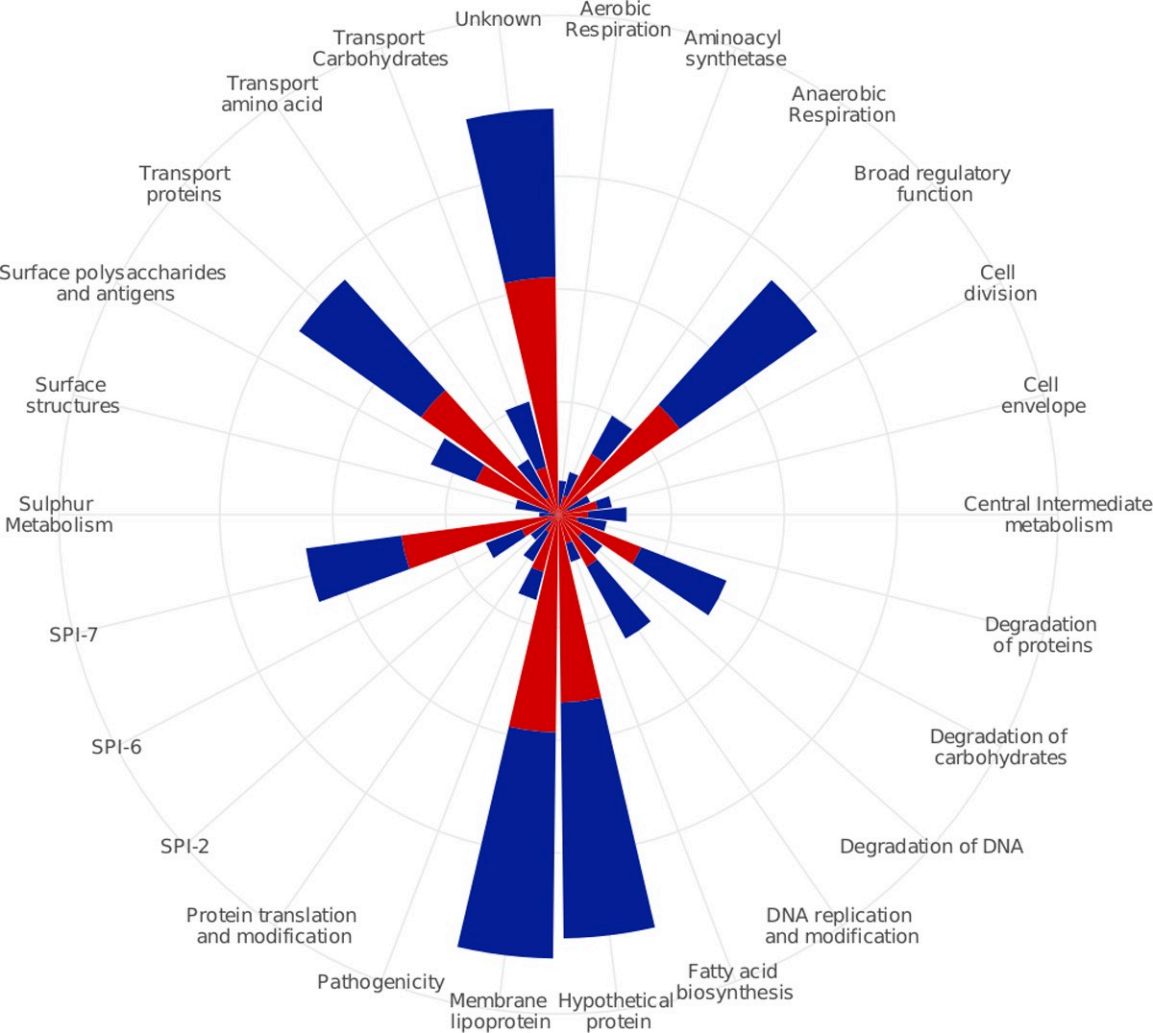
Functional classes of *Salmonella* Typhi genes associated with the highest prevalence of gallbladder-specific nonsynonymous SNPs versus acute-specific nonsynonymous SNPs. Functional classes are shown on the outermost circle. Four circles from the middle represent 5–10–15–20 percent of the cumulative percentage of functional classes. Red and blue blocks are representatives of gallbladder and acute isolates, respectively.

Overall, the gallbladder- and acute-specific NSs across all genotypes could be grouped into 78 functional categories. The highest prevalence of these NSs was found in genes encoding hypothetical proteins, membrane lipoproteins, unknown functions, transport/binding proteins, SPI-7, general regulatory functions, surface polysaccharides and antigens, carbohydrate degradation, and DNA replication/modification. The proportions of NSs in SPI-7, surface polysaccharides and antigens, pathogenicity, cell envelope, anaerobic respiration, fatty acid biosynthesis and transport/binding proteins were all higher in gallbladder than acute isolates (Fig 3). Notably, the data showed that the proportion of NSs in the *viaB* operon (encoding the Vi antigen, target of the typhoid conjugate vaccine (TCV)) was significantly higher in gallbladder isolates compared to the acute isolates across all genotypes (9/228 compared to 7/469, Chi-squared test, *p* = 0.04). Similar results were obtained when considering only *S*. Typhi isolates belonging to subclade 4.3.1, with gallbladder isolates having more specific NSs in the *viaB* operon than the acute isolates (5/60 compared to 1/67, Fisher’s exact test, *p* = 0.09). Additionally, we identified two gallbladder isolates (GB428 and GB003) that had lost the Vi capsular polysaccharide due to the deletion of the entire 134kb SPI-7 region.

### Positive selection associated with typhoid carriage

Finally, we investigated signatures of positive selection by identifying analogous genetic variation detected in different gallbladder isolates. Among the gallbladder specific NSs, a number of different mutations were present in the same gene or the same biological pathway in at least two phylogenetically unlinked gallbladder isolates. For example, within the *viaB* operon, there were two NSs at codon 137 and 462 in the *tviE* gene (isolates GB580 and GB026) and six NSs in codons 166, 504, 506, 508, 665 and 752 in the *tviD* gene (isolates GB005, GB026, GB076, GB125 and GB281, respectively). Both genes facilitate the polymerization and translocation of the Vi capsule [25]. Convergent NSs were also observed in the *rpoS* gene (sigma factor sigma-38) of isolates GB125 (nonsense mutation at codon 247) and GB705 (NSs at codon 94 and 250), which may impact general stress response and nutrient starvation. A further example was NSs at codon 59 and 230 in the *degS* gene (serine protease) (isolates GB005 and GB169). *DegS* is a component of the DegS-DegU two-component regulation system involved in expression of several degradative enzymes for salt stress responses and growth-limiting conditions. Additionally, three isolates (GB005, GB026 and GB705) each possessed an NS (codons 335, 406 and 946, respectively) in STY1242 (*ptsG*—glucose-specific PTS system IIBC component). PtsG enzyme is a component of the glucose-specific phosphotransferase system and plays a role in phosphorylation and translocation of glucose across the bacterial membrane, and is induced in carbon-limited conditions [26]. NSs in several other genes were observed in >2 carriage isolates, including STY0429 (*SbcC*—exonuclease), STY0661 (*dmsC*—molybdopterin containing oxidoreductase membrane anchor subunit), STY1447 (putative ribulose-5-phosphate 3-epimerase) and STY2760 (*ratA*—putative exported protein) (Table 2).

**Table 2:** Nonsynonymous mutations associated with positive selection in gallbladder isolates.

Position in CT18	S/NS	Gene	Product	Functional class	GB005	GB026	GB044	GB076	GB125	GB169	GB199	GB266	GB281	GB335	GB368	GB387	GB441	GB580	GB705
complement (437771‥440875)	NS	STY0429	exonuclease sbcC	degradation of DNA				R394H					L646P						
661366‥662133	NS	STY0661	molybdopterin-containing oxidoreductase	unknown		E5K	V9M												
1196033‥1197466	NS	STY1242	PTS, glucose specific IIBC component	transport carbohydrate	A112T	H136R													G316S
complement (1399142‥1399774)	NS	STY1447	ribulose-5-phosphate 3 epimerase	unknown		E164K							I101V						
complement (2220042‥2221727)	NS	STY2389	two-component system sensor kinase	broad regulatory function									P11L	V49A					
complement (2331373‥2334009)	NS	STY2499	DNA gyrase submit A	DNA replication and function	D97N								L824F						
complement (2915077‥2916069)	NS	rpoS	RNA polymerase sigma subunit RpoS	broad regulatory function			Q246*		W247*										T94P, E250V
complement (2915077‥2916069)	NS	degS	serine protease	degradation of proteins	E230K					V59A									
complement (4516537‥4518273)	NS	tviE	glycosyl transferases	SPI-7		A462T												H137Y	
complement (4519050‥4521545)	NS	tviD	Vi polysaccharide biosynthesis protein	SPI-7	F166L	P752Q, V508I		G506C	R665H				Q504L						

* mutations generating a stop codon

### Evidence of selective pressure on lipopolysaccharide

With respect to convergent mutations within the same biological pathways, there were a number of gallbladder-specific NSs involved in LPS O-antigen synthesis and modification; for example, an NS in the *rfc* gene (regulator of O-antigen polymerization) in isolate GB441, an NS in STY2629 (LPS modification acyltransferase) in isolate GB335, two NSs in *rfbE* (CDP-tyvelose-2-epimerase) and *rfaG* genes (LPS core biosynthesis protein) in isolate GB281, and three NSs in the *rfbK* (phosphomannomutase), *manB* (phosphomannomutase), and *rfaD* genes (ADP-L-Glycero-D-mannoheptose-6-epimease) in isolate GB026. *RfbK* and *manB* are both related to GDP-mannose synthesis for the LPS, and *rfaD* is an enzyme that catalyzes the conversion of ADP-D-glycerol-D-mannoheptose to ADP-L-glycerol-D-mannoheptose, a precursor for the synthesis of inner-core LPS.

## Discussion

As stakeholders consider introduction of TCV into their national immunization programmes, research into the role of chronic carriers in bacterial persistence and disease transmission is needed to forecast the feasibility of typhoid elimination and to inform appropriate public health measures. However, epidemiological investigations of typhoid carriage are challenging, given that this population is difficult to identify and follow. Currently, the environmental factors driving the evolution of *S*. Typhi within the gallbladder are poorly understood and little is known about the adaptive mechanisms that may promote long-term survival. Our relatively small study is the largest genomic investigation of *S*. Typhi gallbladder carriage in a typhoid endemic setting and allowed us to provide unprecedented insight into the genetic and phylogenetic signatures associated with typhoid carriage.

Our data demonstrated, contrary to previous suggestions [27], that carriage of typhoid in the gallbladder was not restricted to any particular genotype and was associated a diverse range of bacterial genotypes, which largely mirrored the genetic structure of the bacterial population causing acute disease in Nepal. Further, typhoid carriage was not confined to specific AMR phenotypes, signifying that carriage is not associated with treatment failure with specific antimicrobials interacting with corresponding AMR profiles. However, by comparing the pairwise SNP distances between gallbladder and acute isolates within the same genotype, we found that gallbladder isolates displayed significantly greater genetic diversity compared to acute isolates, which suggests that long-term exposure to the gallbladder environment results in different accumulated adaptive mutations over time than would be generated in acute isolates. Our phylogenetic reconstruction of *S*. Typhi revealed that a number of gallbladder isolates were located at the tips of atypically long terminal branches, signifying that chronic carriage isolates develop distinct phylogenetic signatures which could be potentially utilized for the identification of organisms arising from chronic carriers. Further investigating this phenomenon, we found that the annual distribution of NPDs of acute isolates, which likely reflects mutation accumulation in the natural environment, was highly comparable across years and could be exploited to disaggregate recent carriers from longer-term carriers. If carriers are relevant for sustaining transmission in endemic area, then we predict that they would play an increasingly important role as acute transmission declines. For example, if environmental transmission decreases through chlorination or acute transmission is controlled through immunisation, transmission via person-to-person transmission via chronic carriers would become proportionally more prevalent. Therefore, the annual NPD distribution could be used to measure the impact of typhoid vaccination on disease transmission dynamics in locations introducing new disease control measures.

The role of chronic carriage in disease transmission represents one of the most long-standing questions in typhoid fever. Though typhoid carriers have been widely considered as an important source of infection, their exact contribution to transmission in endemic areas is not well understood. Previous molecular epidemiological studies in endemic regions highlighted an abundance of long-cycle environmental transmission in these settings, with a wide diversity of co-circulating bacterial genotypes isolated from acute typhoid patients [28–31], suggesting that person-to-person transmission makes a minimal contribution to new typhoid cases in an endemic area. Here, few gallbladder isolates clustered in close proximity or were directly linked with acute isolates and could generally be identified by their placement on long terminal branches. This increase in genetic diversity and the lack of known *S*. Typhi virulence factors (SPI-7) in some carriage isolates, which was not reciprocated in the acute isolates, call into question the role of carriers in disease transmission. Notably, none of the pre-surgical stool cultures from these patients undergoing cholecystectomy were positive for *S*. Typhi. However, the infectivity and transmission fitness of gallbladder isolates must be investigated further, as we cannot rule out the possibility that gallbladder carriage of *S*. Typhi can become a more important reservoir of infection when environmental transmission is successfully reduced. Further, the fact that gallbladder isolates display greater genetic variation than acute isolates implies that the gallbladder may act as an important ecological niche for generating novel genotypes.

By identifying NS mutations occurring specifically in gallbladder isolates and classifying them into predicted functional classes for comparison with those of acute isolates, we found that gene degradation by nonsense mutations was significantly higher in gallbladder isolates compared to acute isolates within subclade 4.3.1. The effects of gene inactivation on phenotype, fitness and adaptation of carriage isolates inside the gallbladder are currently unknown. Further investigation of this phenomenon is necessary, as gene inactivation has been shown to be an important molecular mechanism in human adaptation in the evolutionary history of *S*. Typhi [32,33].

We additionally found evidence for the enrichment of NSs in genes encoding the Vi polysaccharide capsule in gallbladder isolates. The Vi antigen is immunogenic and anti-Vi antibody gradually wanes in acute typhoid patients after recovery, but can be detected in plasma from chronic carriers [34,35]. Data from sero-surveillance studies for chronic carriage have commonly reported elevated anti-Vi antibodies in healthy individuals, which could be associated with carriage or repeated infection [36,37]. Immunofluorescent staining of biofilms produced by *S*. Typhi on the surface of human gallstones demonstrated an abundance of Vi capsule on the surface of the colonising bacteria, suggesting that *S*. Typhi expresses Vi during colonisation of the apical surface of the gallbladder [19]. The increased frequency of nonsynonymous mutations in the viaB operon (*tviB*, *tviD* and *tviE*) of gallbladder isolates, combined with high anti-Vi antibody titres in plasma [38] suggest that *S*. Typhi residing in the gallbladder are under sustained immune pressure. The observation that two gallbladder isolates lacked genes encoding proteins for Vi capsule biosynthesis again suggests that these were subject to selective pressure and that the loss of Vi may be an adaptive mechanism for long-term survival. The generation of Vi-negative *S*. Typhi may also call into question the possibility of their proliferation following mass immunization with TCV.

Identifying genes under selection among gallbladder isolates is crucial for understanding the evolutionary forces and bacterial adaptation to the gallbladder environment during carriage. Signatures of positive selection were detected in a number of genes containing differing gallbladder-specific NS mutations in at least two phylogenetically unlinked gallbladder isolates. Many of these genes are associated with gene regulation under stress and virulence gene expression. For example, the global regulatory gene *rpoS* is responsible for general stress responses and nutrient starvation, and regulates biofilm formation, colonization of Peyer’s patches, persistence in the spleen and the synthesis of Vi [39–41]. The *degS* gene is involved in salt stress responses and growth-limiting conditions; STY1242 (*ptsG*—glucose-specific PTS system IIBC component) is activated during carbon starvation. These observations suggest that *S*. Typhi is exposed to a range of stressors within the gallbladder. Furthermore, genes responsible for LPS biosynthesis were also enriched for NS mutations. LPS is the major component of the outer membrane of Gram-negative bacteria and represents one of the main factors contributing to bile salt resistance [42,43]. LPS is also a key structural component of the biofilm extracellular matrix forming on human gallstones [19,44]. The enrichment of NS mutations in genes involved in LPS biosynthesis and modification leads to structural changes in LPS, which we predict will enhance bile resistance and biofilm formation.

This study has its limitations. The number of gallbladder and acute isolates was relatively small and thus impacts interpretation of the mechanisms underlying the phylogenetic distances between some of the gallbladder isolates and their nearest neighbours. Specifically, our ability to infer associations within uncommon genotypes was limited. Additionally, the identified phylogenetic signature inferred to be associated with carriage was not observed for all gallbladder isolates, due to an underrepresentation in the acutely infected population. Additionally, it was impossible to determine the duration of carriage to confirm our findings, as most typhoid carriers from our study did not recall a history of typhoid [13]. However, our data suggest that the potential duration of carriage within our gallbladder isolates was variable, thus leading to variable terminal branch lengths. Despite these limitations, our study is unique and opens up new possibilities for evaluating associations between gallbladder-specific genetic variation and phenotypic differences to better understand the biology of this infectious disease paradox.

## Conclusions

We conclude that typhoid carriage is not associated with any specific genotype nor driven by AMR phenotypes. However, we show that long-term gallbladder carriage generates genetic variation and results in atypically long phylogenetic branch lengths that could be used to distinguish carriage from acute infection. Further, gene degradation via nonsense mutation is enriched in gallbladder isolates in subclade 4.3.1, potentially reflecting selection for survival in the hostile gallbladder environment. The genetic diversity identified here calls into question the role of typhoid carriers in transmission and suggests that carriage may generate novel genotypes. It remains important that we further investigate the epidemiology, genomics, biology and public health impacts of carriage in parallel to the deployment of disease control and elimination efforts. The role of carriers may become increasingly important as we move toward eradication.

## Methods

### Ethics approval and consent to participate

This study was conducted according to the principles expressed in the Declaration of Helsinki and was approved by the institutional ethical review boards of Patan Hospital, The Nepal Health Research Council and The Oxford University Tropical Research Ethics Committee (OXTREC, Reference number: 2108). All enrollees were required to provide written informed consent for the collection and storage of all samples and subsequent data analysis. In the case of those under 18 years of age, a parent or guardian was asked to provide written informed consent. Consent for publication was incorporated as a component of entrance into the study.

### Sampling

Between June 2007 and October 2010, we conducted a *Salmonella* carriage study at Patan Hospital in Kathmandu[13]. In brief, patients residing in the Kathmandu Valley undergoing cholecystectomy for acute or chronic cholecystitis were enrolled; bile and stool samples from these patients were subjected to microbiological examination. *S*. Typhi were isolated from bile samples taken from these patients (referred to as gallbladder isolates). Additionally, 96 randomly selected *S*. Typhi isolates recovered from the blood of patients with acute typhoid fever residing in the same catchment population within the Kathmandu valley were used for a comparison [24] (referred to as acute isolates) (S1 Table).

### Bacterial isolation and antimicrobial susceptibility testing

Bile and stool were collected from all cholecystectomy patients for culture. Bile was inoculated into equal volumes of Selenite F broth and Peptone broth and incubated at 37°C overnight. Broth was subcultured onto MacConkey agar and Xylene Lysine Deoxycholate (XLD) agar. After overnight incubation at 37°C, the plates were examined for the growth of Gram-negative bacteria and colonies were identified by API20E (bioMerieux, France). *S*. Typhi/*S*. Paratyphi A were confirmed by slide agglutination using 02, 09, and Vi antisera (Murex Biotech, Biotech, England).

For the acute isolates, 5–10 ml of blood was taken from all patients with a clinical suspicion of typhoid fever and inoculated into media containing tryptone soya broth and sodium polyanethol sulphonate (up to 25mL). Blood culture bottles were incubated for up to seven days, with blind sub-cultures at 24 hours, 48 hours, and 7 days, or when the broth became cloudy on sheep blood, chocolate, and MacConkey agar. Presumptive *Salmonella* colonies were identified as above.

Antimicrobial susceptibility testing was performed by the modified Bauer-Kirby disc diffusion method with zone size interpretation based on CLSI guidelines [45]. Etests® were used to determine MICs following the manufacturer's recommendations (bioMérieux, France). Ciprofloxacin MICs were used to categorise *S*. Typhi isolates as susceptible (≤0.06 μg/mL), intermediate (0.12–0.5 μg/mL) and resistant (≥1 μg/mL) following CLSI guidelines [45].

### Vi agglutination assay

Two gallbladder isolates of *S*. Typhi (GB003 and GB428) that lacked the Vi polysaccharide biosynthesis (*viaB*) operon were grown on LB agar plates supplemented with increasing concentrations (1mM, 85mM and 170mM) of NaCl. Vi agglutinations were performed on microscope slides by mixing 10μl of single colony suspensions with 50μl of Vi antisera (Murex Biotech, Biotech, England). Agglutination was recorded after gently agitating the slide for 1 minute. Two gallbladder isolates of *S*. Typhi (GB125 and GB169) containing the *viaB* operon were used as controls.

### Whole genome sequencing and SNP analyses

Total genomic DNA from acute and gallbladder *S*. Typhi isolates was extracted using the Wizard Genomic DNA Extraction Kit (Promega, Wisconsin, USA) (S1 Table). 50ng of genomic DNA was subjected to library preparation using the Nextera DNA library prep kit; whole genome sequencing (WGS) was performed on an Illumina MiSeq platform following the manufacturer’s recommendations to generate 250bp paired end reads.

Single nucleotide polymorphisms (SNPs) were called using previously described methods[46]. Briefly, all reads were mapped to the reference sequence of *S*. Typhi strain CT18 (Accession no: AL513382), plasmid pHCM1 (AL513383) and pHCM2 (AL513384) using SMALT (version 0.7.4). Candidate SNPs were called against the reference sequence using SAMtools and filtered with a minimal mapping quality of 30 and a quality ratio cut-off of 0.75. The allele at each locus in each isolate was determined by reference to the consensus base in that genome. This process was performed using *samtools mpileup* and by removing low confidence alleles with consensus base quality ≤20, read depth ≤5 or heterozygous base calls. SNPs in phage regions, repetitive sequences or recombinant regions were excluded, [47,48] which resulted in a final set of 2,186 chromosomal SNPs. SNPs were subsequently annotated using the parseSNPTable.py script in the RedDog pipeline (https://github.com/katholt/RedDog). From the identified SNPs in *S*. Typhi genomes, a subset of 68 were used to assign *S*. Typhi isolates to previously defined lineages according to the existing extended *S*. Typhi genotyping framework [49].

To identify the potential function of genes containing key SNPs, we investigated the known or predicted functions of the identified genes. We identified SNPs occurring exclusively in either acute or gallbladder isolates and genes containing these SNPs were grouped by their predicted or known function based on the *S*. Typhi functional classification scheme developed by the Sanger Institute (www.sanger.ac.uk) using the genome annotation of *S*. Typhi CT18 [50].

The antimicrobial resistance (AMR) gene and plasmid contents of *S*. Typhi isolates were determined using a local assembly approach with ARIBA (Antimicrobial Resistance Identifier by Assembly) [51]. Resfinder [52] and Plasmidfinder [53] were used as reference databases of antimicrobial resistance genes and plasmid replicons, respectively.

### Phylogenetic analyses and pairwise SNP distance

A maximum likelihood phylogenetic tree was reconstructed from the SNP alignment of 120 *S*. Typhi isolates (an *S*. Paratyphi A isolate was included as an outgroup) using RAxML (version 8.2.8) with the generalized time-reversible model and a Gamma distribution to model the site-specific rate variation (GTR+Г). Support for the maximum likelihood (ML) tree was assessed via bootstrap analysis with 1,000 pseudoreplicates. Pairwise phylogenetic distances depicting the phylogenetic branch length separating each pair of taxa within subclade 4.3.1 (H58) were estimated using the function *cophenetic* in the ape package (v4.1) in R (v3.3.2). Phylogenetic distances between each taxon and its nearest neighbour on the phylogenetic tree of subclade 4.3.1 were plotted using ggplot2. To investigate the phylogenetic structure of acute and gallbladder *S*. Typhi isolates from Nepal in the global context, a second maximum likelihood tree was inferred from a separate alignment of 23438 SNPs identified from 120 Nepali *S*. Typhi along with 1820 globally representative *S*. Typhi described previously [54]. A *S*. Paratyphi A isolate was included as an outgroup to root the tree. Support for this ML tree was assessed via 100 bootstrap replicates.

Pairwise genetic distances (the difference in the number of SNPs) within and between acute and gallbladder *S*. Typhi isolates were estimated from the SNP alignment using the ape (v4.1) and adegenet (v2.0.1) packages in R (v3.3.2). Pairwise SNP distances were extracted and plotted using the function *pairDistPlot* in the adegenet package. The Wilcoxon rank-sum test was used for testing the difference in the average pairwise SNP distances between groups.

The raw sequence data generated from this study are available in the European Nucleotide Archive (ENA) under the accession numbers described in S1 Table.

## Supporting information

S1 Table Gallbladder and acute *Salmonella* Typhi isolates and their associated metadata. (XLS)

S1 Fig Phylogenetic structure of acute and gallbladder *Salmonella* Typhi isolates from Nepal in the global context. (TIF)

S2 Fig Distribution of pairwise SNP distances within and between gallbladder (GB) and acute isolates belonging to subclades 4.3.1 (H58) and 3.3.0 (H1). (TIF)
