# Supplementary figures and images for: Gallbladder carriage generates genetic variation and genome degradation in Salmonella Typhi
Source: PLoS Pathog. 2020 Oct 21;16(10):e1008998. doi: 10.1371/journal.ppat.1008998 (PMC7605710; doi:10.1371/journal.ppat.1008998)

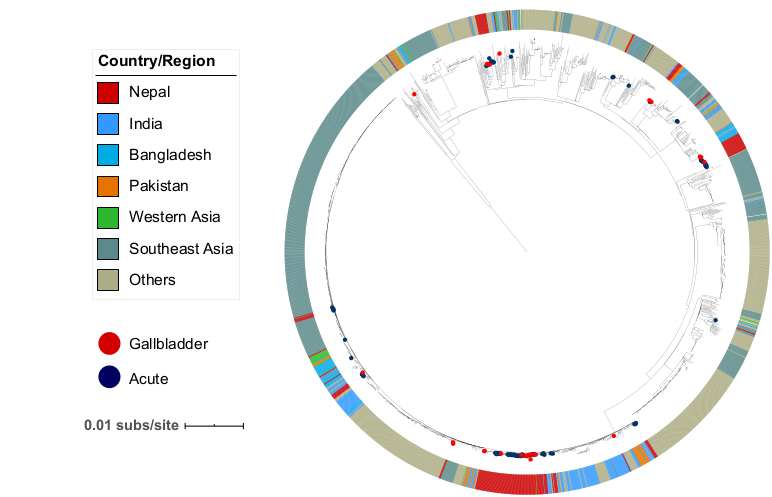

Supplement: S1 Fig — (TIF) [file ppat.1008998.s002.tif]

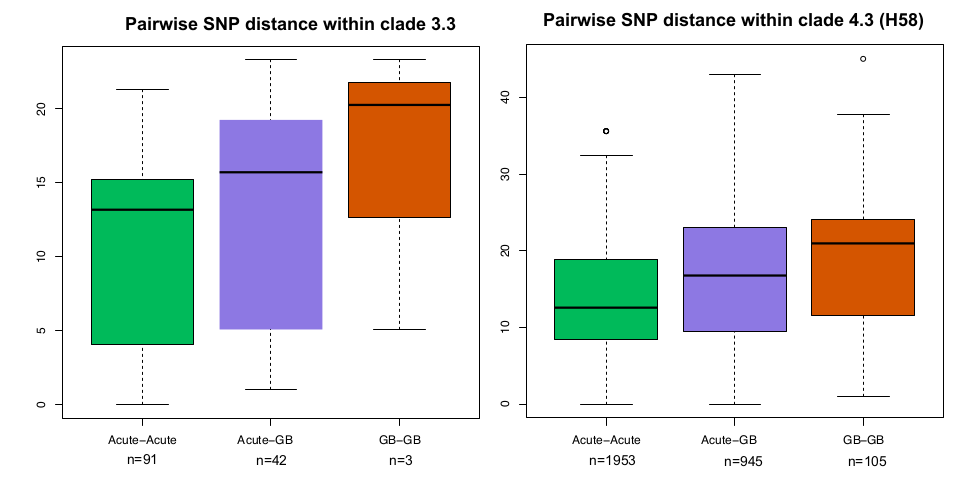

Supplement: S2 Fig — (TIF) [file ppat.1008998.s003.tif]
